# PET/ZnO@MXene-Based Flexible Fabrics with Dual Piezoelectric Functions of Compression and Tension

**DOI:** 10.3390/s23010091

**Published:** 2022-12-22

**Authors:** Yanlu Chen, Xinxin Pu, Xinyu Xu, Menghan Shi, Hui-Jun Li, Ding Wang

**Affiliations:** School of Materials and Chemistry, University of Shanghai for Science & Technology, Shanghai 200093, China

**Keywords:** flexible pressure sensors, MXene, pressure-sensitive, ZnO nanoarrays

## Abstract

The traditional self-supported piezoelectric thin films prepared by filtration methods are limited in practical applications due to their poor tensile properties. The strategy of using flexible polyethylene terephthalate (PET) fabric as the flexible substrate is beneficial to enhancing the flexibility and stretchability of the flexible device, thus extending the applications of pressure sensors. In this work, a novel wearable pressure sensor is prepared, of which uniform and dense ZnO nanoarray-coated PET fabrics are covered by a two-dimensional MXene nanosheet. The ternary structure incorporates the advantages of the three components including the superior piezoelectric properties of ZnO nanorod arrays, the excellent flexibility of the PET substrate, and the outstanding conductivity of MXene, resulting in a novel wearable sensor with excellent pressure-sensitive properties. The PET/ZnO@MXene pressure sensor exhibits excellent sensing performance (S = 53.22 kPa^−1^), fast response/recovery speeds (150 ms and 100 ms), and superior flexural stability (over 30 cycles at 5% strain). The composite fabric also shows high sensitivity in both motion monitoring and physiological signal detection (e.g., device bending, elbow bending, finger bending, wrist pulse peaks, and sound signal discrimination). These findings provide insight into composite fabric-based pressure-sensitive materials, demonstrating the great significance and promising prospects in the field of flexible pressure sensing.

## 1. Introduction

With the development of flexible wearable devices, the demand for multifunctional pressure sensors has increased dramatically. The flexible pressure-sensitive devices based on flexible substrates and sensitive materials possess several unique advantages including high durability, scalability, and portability, thus exhibiting numerous potential applications in the fields of intelligent sensing, physiological activity monitoring, and health diagnosis [1,2,3,4,5,6,7]. Traditional self-supporting flexible films’ poor flexibility, tensile strength, and wear resistance make it difficult for them to meet practical requirements [8,9,10,11]. Thus, it remains a challenge to endow the fabric with excellent pressure sensitivity while being both flexible and breathable. To achieve high detection sensitivity and a wide range of stress/strain detection at the same time, it is crucial to design and construct flexible sensing materials with outstanding sensing capabilities.

To design flexible piezoelectric sensors with high sensitivity, three factors need to be taken into account, which are the species of flexible substrates and sensitive materials and the design of device structures. Textiles used in wearable electronics for medical and human activity sensing should be hygroscopic, soft, breathable, and comfortable against human skin. In the choice of a flexible substrate, PET (polyethylene terephthalate) fabric demonstrates several advantages including reasonable costs, stretchability, and thermal stability up to 100 °C. Other characteristics such as excellent flexibility, breathability, and comfort also attract great attention [12,13]. For the sensitive unit, materials with excellent stability, conductivity, and piezoelectricity are optimal, including graphene [14,15], metal oxides [16], MXene [17,18,19,20], MOF [21,22,23], etc. As a typical semiconductor material, ZnO nanorods with a hexagonal structure benefit from inexpensive preparation costs, great chemical stability, and excellent piezoelectric properties, and have been intensively studied in flexible piezoelectric sensors [24,25]. However, it has been reported that if the ZnO nanorod arrays are assembled on the PET surface, the contact resistance between the ZnO nanorods would be increased, resulting in a high resistance [26]. Therefore, it is urgent to improve the electrical conductivity of the system for further practical applications. Due to its extensive physicochemical features and superior electrical conductivity, Ti_3_C_2_T_x_ (MXene) has received extensive attention in the field of piezoelectric sensors. In previous works, several MXene-based pressure sensors have been constructed as flexible, self-supporting membranes [27,28]. However, its flexibility, tensile properties, and wear resistance limit its performance [29,30]. Therefore, it is envisaged that the combination of PET and MXene to fabricate a flexible substrate could promote electrical conductivity while solving the problem of poor toughness of MXene self-supporting membranes. Furthermore, the excellent piezoelectric properties of ZnO nanorod arrays would provide composite membranes with dual functional properties of compression and stretching.

In this work, the PET/ZnO@MXene composite flexible fabric is prepared by uniformly assembling ZnO nanorod arrays on the surface of the PET fabric. The composites are then coated with MXene nanosheets obtained from etching via the impregnation method. Based on the SEM, XRD, XPS, and other characterization methods, the morphology and structure of the sensor are analyzed. The pressure-sensing performance of the composite fabric sensor is tested, including sensitivity, response recovery curve, tensile resistance, and human signal detection. Finally, the flexible wearable device is made to conduct preliminary exploration and experimental verification for its application scenario.

## 2. Materials and Methods

### 2.1. Materials

Lithium fluoride (LiF, 99.9%), hydrochloric acid (HCl, 37%), anhydrous zinc acetate (CH_6_O_4_Zn, 99.5%), polyetherimide (PEI, RG), methanol (CH_3_OH, 99.9%), and acetone (C_3_H_6_O, 99.9%) were purchased from Adamas. The Ti_3_AlC_2_ MAX phase (400 mesh) was purchased from 11 Technology Co. Ltd. (Jilin, China). Hexamethyleneimine (C_6_H_13_N, 98%) and zinc nitrate hexahydrate (Zn(NO_3_)_2_·6H_2_O, AR) were purchased from Aladdin reagents. All chemicals were used as purchased without further purification.

### 2.2. Synthesis of MXene Nanosheets

To synthesize Ti_3_C_2_T_x_ nanosheets from the Ti_3_AlC_2_ MAX phase, a reported etching method was utilized based on the etching solution of LiF/HCl [31]. Firstly, 2 g of LiF and HCl (20 mL, 9 M) were stirred for 30 min to ensure that LiF was entirely dissolved. Then the solution was heated to 35 °C, and the Al layer in the precursor MAX phase was slowly etched at a certain speed for 24 h. The resulting solution was repeatedly washed with deionized water until the pH value was higher than 6. Anhydrous ethanol (40 mL) used as an intercalating agent was added to the solution and the mixture was sonicated for 1 h. The solution was then centrifuged at 10,000 rpm for 10 min and the sediment was collected. The precipitate was mixed with deionized water (20 mL) under ultrasonication for 20 min, and further centrifuged at 3500 rpm for 3 mins. The black-brown upper solution was collected, which was the MXene few-layer nanosheets. Using repeated centrifugation, the yield of MXene nanosheets could be increased.

### 2.3. Fabrication of Flexible Pressure Sensor Based on PET/ZnO@MXene

**Scheme 1:**
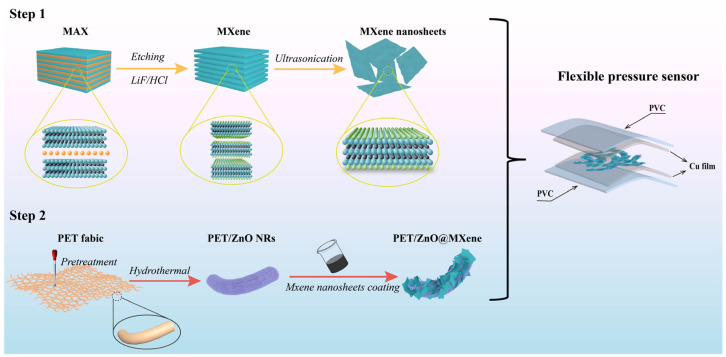
Synthesis diagram of the PET/ZnO@MXene composite fabric.

Large pieces of fabric were cut into 2 cm × 2 cm pieces and treated ultrasonically in methanol, acetone, and deionized water solutions for 10 min to obtain a clean PET flexible fabric. Then 0.02 g of zinc acetate dihydrate was dissolved in 20 mL of anhydrous ethanol and stirred at 600 rpm for 30 min to form a transparent solution. One milliliter of the zinc acetate solution was added to the flexible PET fabric base and dried in the atmosphere. This adding–drying step was repeated 4–5 times. The as-made flexible material was then treated at 350 °C for 20 min. Meanwhile, zinc nitrate hexahydrate (0.745 g), polyetherimide (PEI, 0.4 g), and hexamethylenetetramine (HMTA, 0.35 g) were dissolved in 100 mL of deionized water and stirred at 600 rpm for 30 min. The pre-treated PET fabric material was then inverted in the configured growth solution and hydrothermally reacted for 4 h at 90 °C. The obtained PET/ZnO NRs flexible fabric was repeatedly dipped into the 1 g/mL of MXene nanosheets aqueous solution, and this step was repeated 5 times to obtain a uniformly coated PET/ZnO@MXene fabric. The copper films were attached as electrodes to both ends of the PET/ZnO@MXene flexible fabric, and two clean flexible PVC films covered the two ends of the pressure-sensitive device to prevent material contamination and ensure the stability of the equipment during the test. The picture of the fabricated sensor is shown in Figure S1. Scheme 1 is the synthesis diagram of the PET/ZnO@MXene composite fabric.

### 2.4. Characterization

The crystalline structures of PET, MXene, PET/ZnO NRs, and PET/ZnO@MXene were characterized by X-ray diffraction (XRD, D8-Advance, Saarbruecken, Germany) with 2θ in the ranges of 5~60° at room temperature. The morphology of the PET, PET/ZnO NRs, and PET/ZnO@MXene film was observed with field emission scanning electron microscopy (FE-SEM, JEOL JSM-7001F, Tokyo, Japan). The surface compositions and chemical states were examined by X-ray photoelectron spectroscopy (XPS, VG ESCALAB 210, Massachusetts, USA). The sensing performance was measured by the Flexible Device Analysis System (AES-4SD-SA7102, SINO AGGTECH, Beijing, China).

## 3. Results and Discussion

### 3.1. Morphology and Structure Characterization

**Figure 1:**
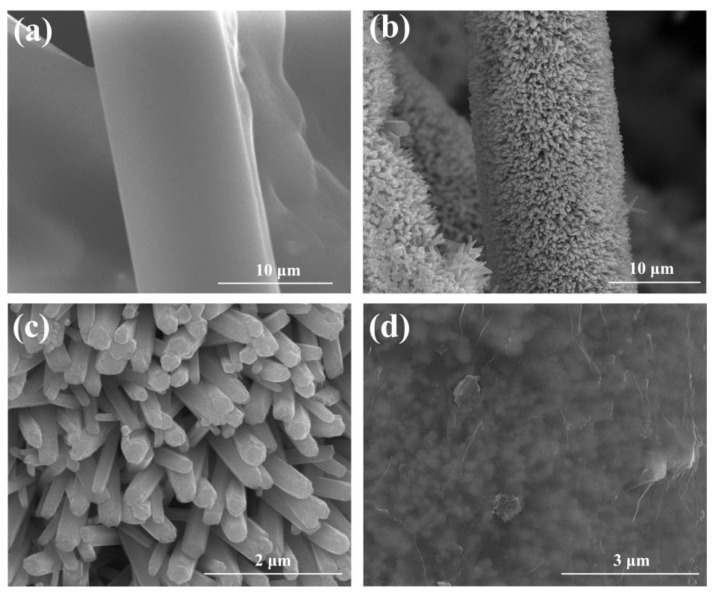
SEM images of the obtained materials. (**a**) PET fabric, (**b**) PET/ZnO composite fabric, (**c**) magnified image of the PET/ZnO composite fabric, and (**d**) PET/ZnO@MXene composite fabric.

The SEM images of PET, PET/ZnO, and PET/ZnO@MXene are displayed in Figure 1. The PET fiber showed a smooth surface with a diameter of approximately 10 μm (Figure 1a and Figure S2). After hydrothermal processing, uniform and dense ZnO nanorod arrays were found to grow on the surface of PET, shown in Figure 1b. The high-resolution SEM image (Figure 1c and Figure S3) showed that the aligned ZnO arrays were hexagonal nanorods with a diameter smaller than 300 nm. According to the diameter change of the PET before and after ZnO growth, the length of the nanorod was estimated to be approximately 2 μm. After being impregnated with MXene nanosheets, the as-made PET/ZnO fabric displayed a rough and wrinkled MXene film on the surface in Figure 1d and Figure S4. The tight encapsulation of MXene nanosheets on the ZnO surface might be owing to the electrostatic interactions between the negatively charged hydrophilic MXene nanosheets and oxides, which is beneficial to promote electron transmission [32].

**Figure 2:**
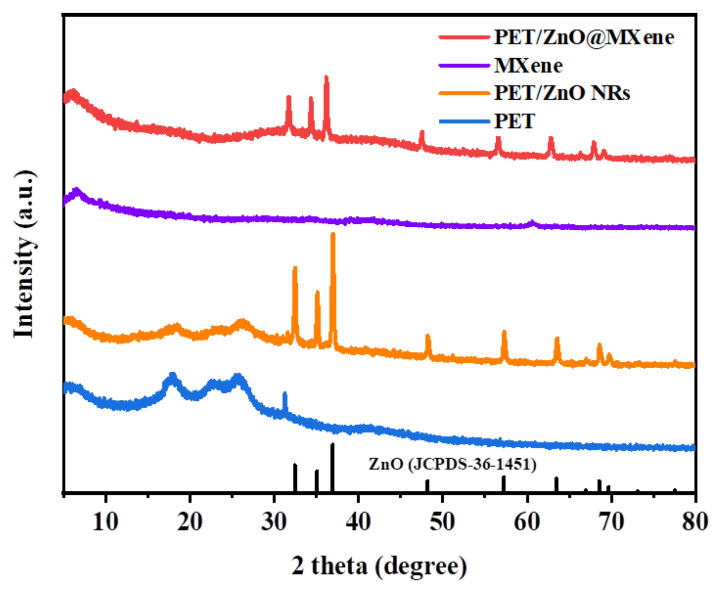
XRD patterns of PET, MXene, PET/ZnO NRs, and PET/ZnO@MXene composite fabrics.

The structures of the PET, PET/ZnO, and PET/ZnO@MXene composite fabrics were characterized by XRD. In Figure 2, the diffraction peaks at 2θ = 18.1°, 23.6°, and 25.9° are attributed to the monoclinic crystal structure of PET. For the PET/ZnO NRs, the diffraction peaks at 2θ = 32.4°, 35.2°, 37.0°, 48.3°, 57.3°, 63.6°, and 68.6° could be owing to that of hexagonal wurtzite ZnO, matching JCPDS NO. 36-1451 [33]. The existence of MXene in the PET/ZnO@MXene fiber fabric was proven by the (002) diffraction peak at 2θ = 7.0° [32,34]. Since the surface of PET was covered by ZnO nanorod arrays and Mxene, the diffraction peaks of PET could not be observed in the PET/ZnO@MXene composite. No additional diffraction peaks of impurities were shown in the XRD pattern, demonstrating a relatively high purity of the developed sensitive device.

**Figure 3:**
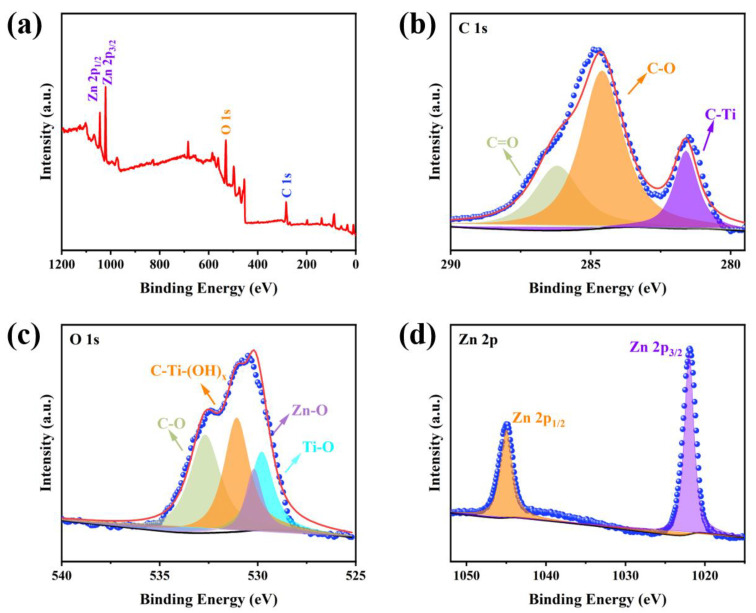
XPS spectra of PET/ZnO@MXene composite fabric, (**a**) full-survey XPS spectrum, (**b**) C 1s, (**c**) O 1s, and (**d**) Zn 2p high-resolution spectrum.

The chemical states and elemental compositions of PET/ZnO@MXene were analyzed by XPS. Shown in the full-survey XPS spectrum of PET/ZnO@MXene, the presence of Zn, C, O, Ti, and F elements is confirmed. Furthermore, the high-resolution spectra of the C, O, and Zn elements were analyzed. In Figure 3b, strong peaks with the binding energy of approximately 286.2, 284.6, and 281.6 eV could be attributed to C-O/C-N, C-C, and C-Ti bonding, respectively [34]. The high-resolution XPS spectrum of O 1s can be divided into four distinct peaks in Figure 3c, with binding energies of 532.7 eV, 531.1 eV, 530.2 eV, and 529.8 eV. The four peaks corresponded to the presence of C-O, C-Ti-(OH)_x_, Zn-O, and Ti-O, respectively [35,36,37]. The high-resolution XPS spectrum of Zn 2p shown in Figure 3d displayed two fitted peaks at 1044.2 eV and 1021.1 eV, which were assigned to Zn 2p_1/2_ and Zn 2p_3/2_, respectively. These results indicate that on the surface of ZnO NRs, the chemical compound valence of Zn is in the positive divalent oxidation state [33,36,38].

### 3.2. Pressure Sensing Performance of the PET/ZnO@MXene Composite Fabric

**Figure 4:**
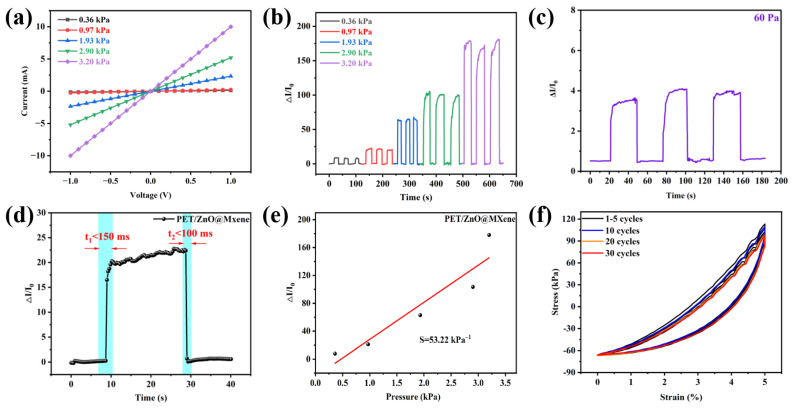
Electronic and sensing performance of a PET/ZnO@MXene flexible composite fabric pressure sensor. (**a**) I-V curve, (**b**) I-T curve under different applied pressures, (**c**) response change under P = 60 Pa pressure, (**d**) response recovery time under P = 0.97 kPa pressure, (**e**) linear sensitivity fitting diagram within the pressure ranges of 0.36–3.20 kPa, (**f**) strain–stress curve after 30 cycles under the strain of 5%.

The developed pressure sensors can be used to track weak vibration or bending signals based on their sensitivity to minor deformation. We examined the sensor’s pressure-sensing capability towards various pressures. In Figure 4a, the I-V curves under different pressures showed that the sensor’s response to static pressure was stable (–1~1 V). The I-V curves exhibited good linear Ohmic contact behavior, with the resistance value (slope of the I-V curve) remaining constant under the applied pressures of 0.36, 0.97, 1.93, 2.90, and 3.20 kPa. The I-V curve’s slope increases as the strain increases, implying that the resistance of the composite increases as the pressure increases. In the following tests, the voltage value was set as 1 V. In Figure 4b, the sensor exhibited an obvious response under different strains, and the response value increased with the increase in strain. Additionally, the dynamic response and recovery curves of the flexible pressure sensor based on PET/ZnO@MXene achieved almost the same response value within five measurement periods under each strain, indicating that the sensor has good recycle stability. In addition, a clearly discernable current change signal appeared even under the pressure of 60 Pa as shown in Figure 4c, indicative that the flexible pressure sensor based on the composite fabric has a low detection limit.

Response and recovery speed is also an important parameter for electronic skin strain units used in human health monitoring. Since certain physiological signals (pulse, heart rate, respiration, etc.) range in frequencies from 0.2 to 2.0 Hz, fast recognition speed is critical for precise sensing. Figure 4d shows a single response and recovery process of the PET/ZnO@MXene flexible pressure sensor under a stress condition of 0.97 kPa. The response and recovery times were as short as 150 ms and 100 ms, respectively, which demonstrated the excellent response and recovery characteristics of the sensor. Generally, the sensitivity of a pressure sensor is defined as S = δ (ΔI/I_0_)/δp, where ΔI is the relative change in current, I_0_ is the current without applied pressure, and P is the applied pressure. Using sensitivity fitting, as shown in Figure 4e, the sensitivity S was calculated to be 53.22 kPa^−1^. Notably, the fabric is stretchable since the framework structure of the composite fabric can be extended in different directions. Therefore, a uniaxial tensile test was conducted to evaluate the mechanical property of the flexible sensor. After 30 repeated cycles of stretching under strain (ε) of 5%, the device showed stable mechanical scalability. After cyclic loading, the sensitivity change of the device was negligible. Based on the above results, the stable performance of the textile sensor provides feasibility in long-term service. Table S1 shows the comparison of different flexible sensing parameters between our sensor and previously reported works. The sensitivity value of our sensor exceeded several sensors and is much more sensitive than the reported PET/MXene film. The response times are also competitive [39,40,41,42]. Moreover, the minimum pressure value of 60 Pa is much lower than other flexible sensing materials.

### 3.3. Wearable Testing Based on PET/ZnO@MXene Composite Fabric

**Figure 5:**
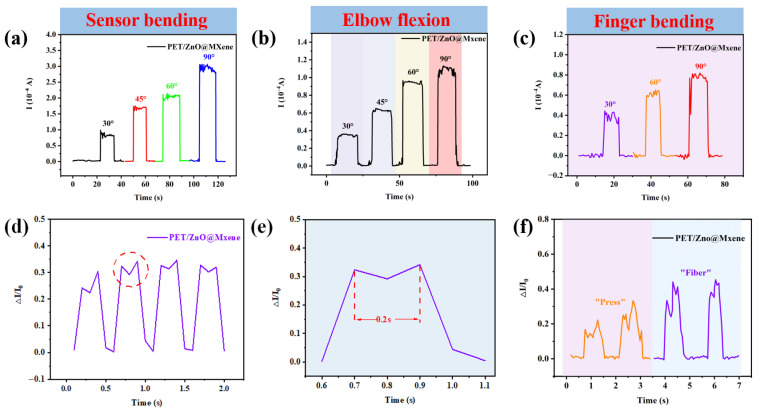
Current response curve (**a**) when pure device is bent, (**b**) when elbow joint is bent, (**c**) when finger is bent, (**d**) of pulse vibration. (**e**) pulse amplification curve of a single period, and (**f**) vocal cord vibration curve.

Textile pressure sensors with high sensitivity and flexibility can be used to detect different degrees of device bending, finger gestures, elbow bending, and acoustic vibration. Considering that human motions might trigger different bending phenomena, a series of tests were conducted to verify whether the sensor can meet our daily monitoring needs. As shown in Figure 5a, the sensor can sensitively identify the bending of different angles. Obvious current signal changes appear at 30°, 45°, 60°, and 90°, and the bending angle is positively correlated with the response. Furthermore, the flexible fabric pressure sensor is attached to the skin of the elbow joint. When the elbow was bent, the contact area between the copper electrodes and the inner MXene-coated fabric increased, resulting in more conductive paths along the MXene nanosheets. This led to a corresponding increase in the current (see Figure 5b). For finger bending, the signal can also be quickly recognized as shown in Figure 5c. In addition to the bending force, the pressure sensor can be used to detect small pressure fluctuations, such as pulse signals collection, which is significant in blood pressure and certain cardiac function diagnoses [23]. In Figure 5d, the radial pulse of the wrist was a reflection of the heart rate, and the unique waveform of each cycle pulse in a single-cycle pulse magnification consisted of two characteristic peaks, namely P_1_ (systolic wave) and P_2_ (diastolic wave) peaks. The repeated discernable peaks reflect the high sensitivity and fast response rate of the sensor.

The flexible pressure sensor can also be used to detect acoustic vibrations due to its high sensitivity. To demonstrate this capability, the sensors were attached to membranes and placed close to the vocal cords. Figure 5f shows that the textile sensors attached to the neck skin could non-invasively monitor the pressure differences in muscle movements during speech. When we said different words such as “Press” and “Fiber”, the device showed high sensitivity and distinct current curves. Therefore, strain sensors demonstrate great potential in the field of health monitoring and human–computer interaction. It should be noted that the strain sensor deformation degree in the process of motion monitoring and physiological signal detection is more reasonable than that in the process of standard strain sensing performance measurement.

## 4. Conclusions

In conclusion, a unique flexible pressure sensor based on PET/ZnO@MXene was prepared through hydrothermal and impregnation methods. The XRD, SEM, and XPS characterizations confirm that ZnO nanorod arrays are assembled uniformly with high density on the surface of the PET fiber, while the MXene nanosheets are coated or interspersed on the surface of the ZnO nanorod to regulate the electron transport characteristics. Due to the excellent piezoelectric properties of ZnO and electron transport characteristics of MXene, the flexible pressure sensor based on the composite fabric achieves high sensitivity (53.22 kPa^−1^), fast response recovery time (<150 ms), and excellent bending stability (over 30 cycles under the strain of 5%). The sensor is further used as a part of human skin or clothing to detect various external pressures, human activities, and even real-time pulse wave monitoring. Based on the characteristics of low-cost manufacturing, high performance, flexibility, and human friendliness, this work proposes a stretchable pressure sensor with great promising potential in smart textiles or wearable electronics.

## Data Availability

No new data were created or analyzed in this study. Data sharing is not applicable to this article.

## Supplementary Materials

The following supporting information can be downloaded at: https://www.mdpi.com/article/10.3390/s23010091/s1, Figure S1: A physical image of the preparation process and sensing test. (a) PET, (b) small pieces of PET, (c) PET/ZnO, (d) MXene solution (the enlarged illustration is PET/ZnO@MXene), (e) fabrication of flexible pressure sensor, (f) the sample placed on a flexible test platform; Figures S2–S4: SEM images of PET fibers, PET/ZnO, PET/ZnO@MXene in different multiples. (b) is the enlarged view of the red box in (a), (c) is the enlarged view of the red box in (b), and (d) is the enlarged view of the red box in (c). Table S1: Comparison of different flexible sensing parameters between our sensor and some previously reported works. Refs. [39,40,41,42] are cited in supplementary materials.
