# Supplementary material for: PET/ZnO@MXene-Based Flexible Fabrics with Dual Piezoelectric Functions of Compression and Tension
Source: Sensors (Basel). 2022 Dec 22;23(1):91. doi: 10.3390/s23010091 (PMC9823752; doi:10.3390/s23010091)
Supplement: Supplementary file 1 [file sensors-23-00091-s001.zip › sensors-2109257-supplementary.pdf]

Supporting information

PET/ZnO@MXene-based Flexible Fabrics with Dual Piezoelectric Functions of Compression and Tension

Yanlu Chen[1,†], Xinxin Pu[1,†], Xinyu Xu[1], Menghan Shi[1], Hui-Jun Li[1,*], Ding Wang[1]

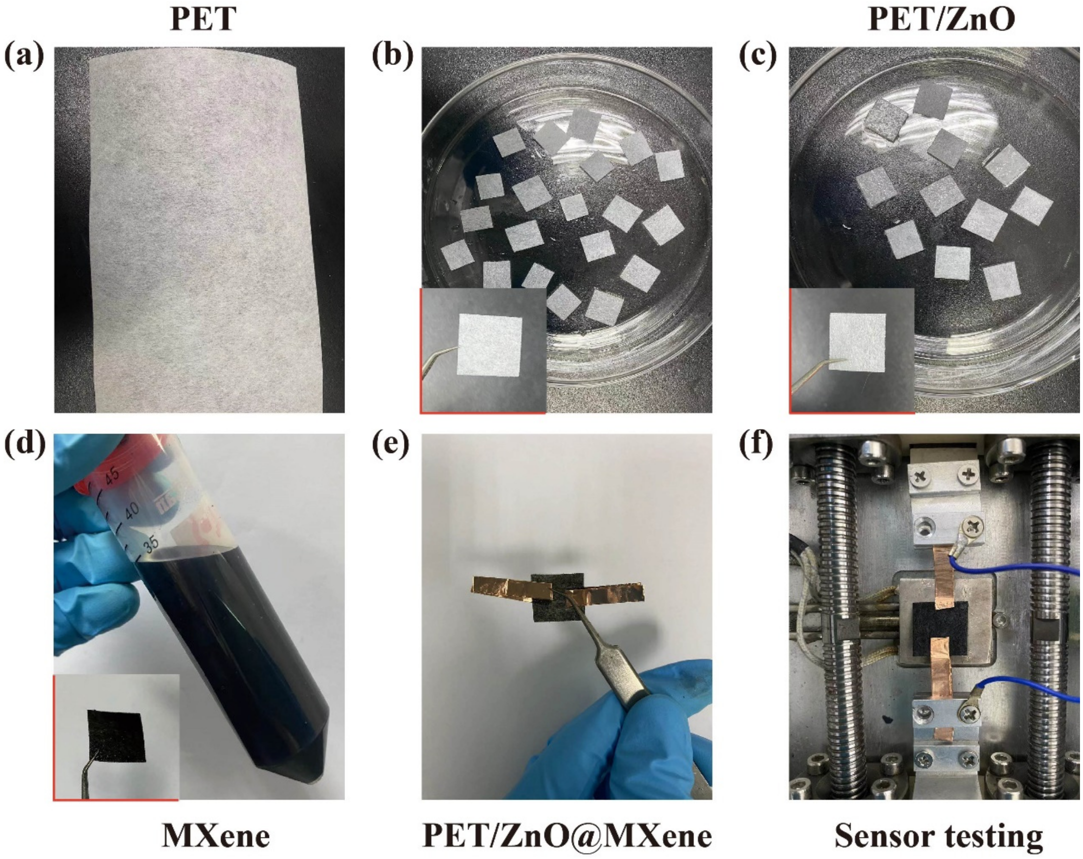

Figure S1. A physical image of the preparation process and sensing test. (a) PET, (b) small pieces of PET, (c) PET/ZnO, (d) MXene solution (the enlarged illustration is PET/ZnO@MXene), (e) fabrication of flexible pressure sensor, (f) the sample placed on a flexible test platform.

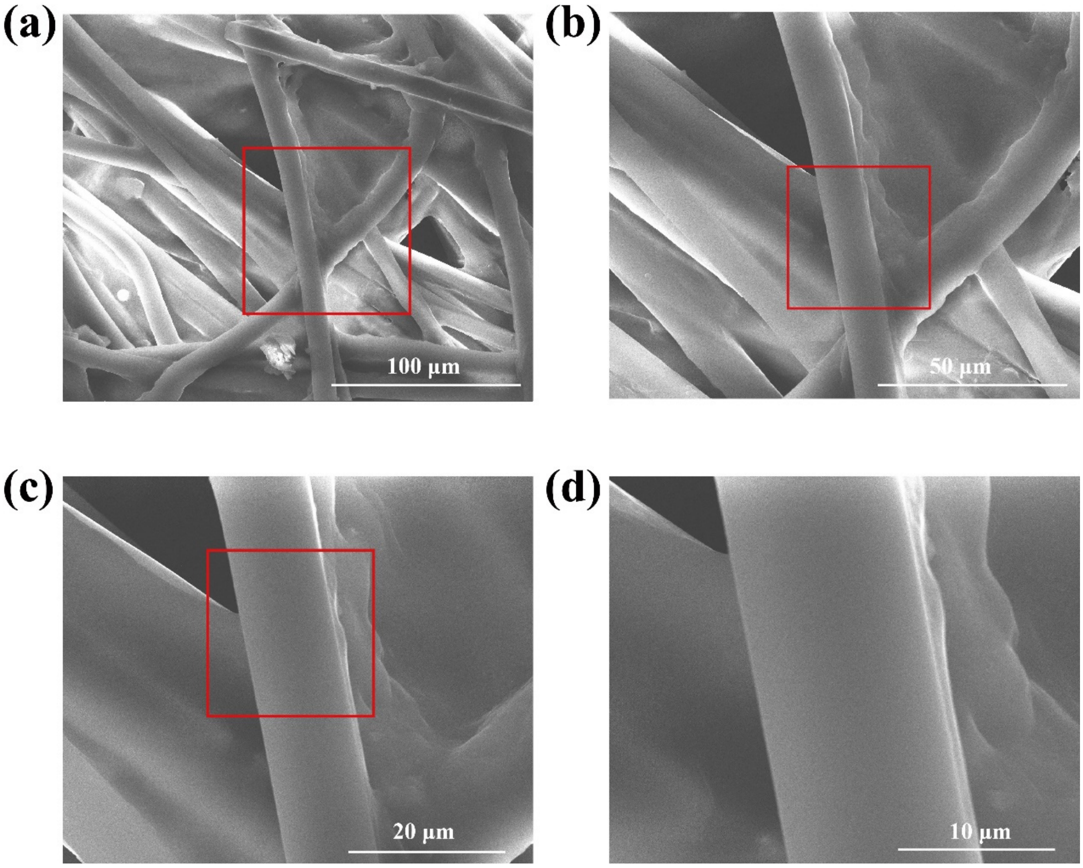

Figure S2. SEM images of PET fibers in different multiples (the red box is the enlarged part). (b) is the enlarged view of the red box in (a), (c) is the enlarged view of the red box in (b), and (d) is the enlarged view of the red box in (c).

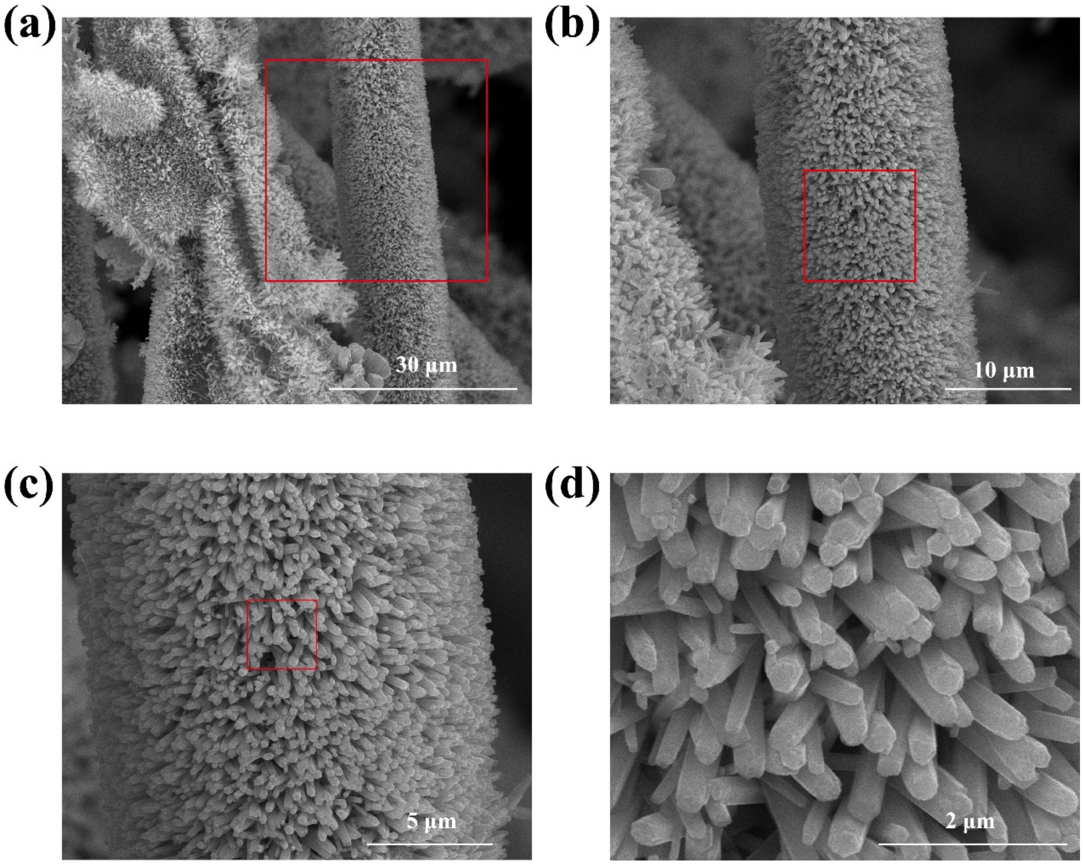

Figure S3. SEM images of PET/ZnO in different multiples (the red box is the enlarged part). (b) is the enlarged view of the red box in (a), (c) is the enlarged view of the red box in (b), and (d) is the enlarged view of the red box in (c).

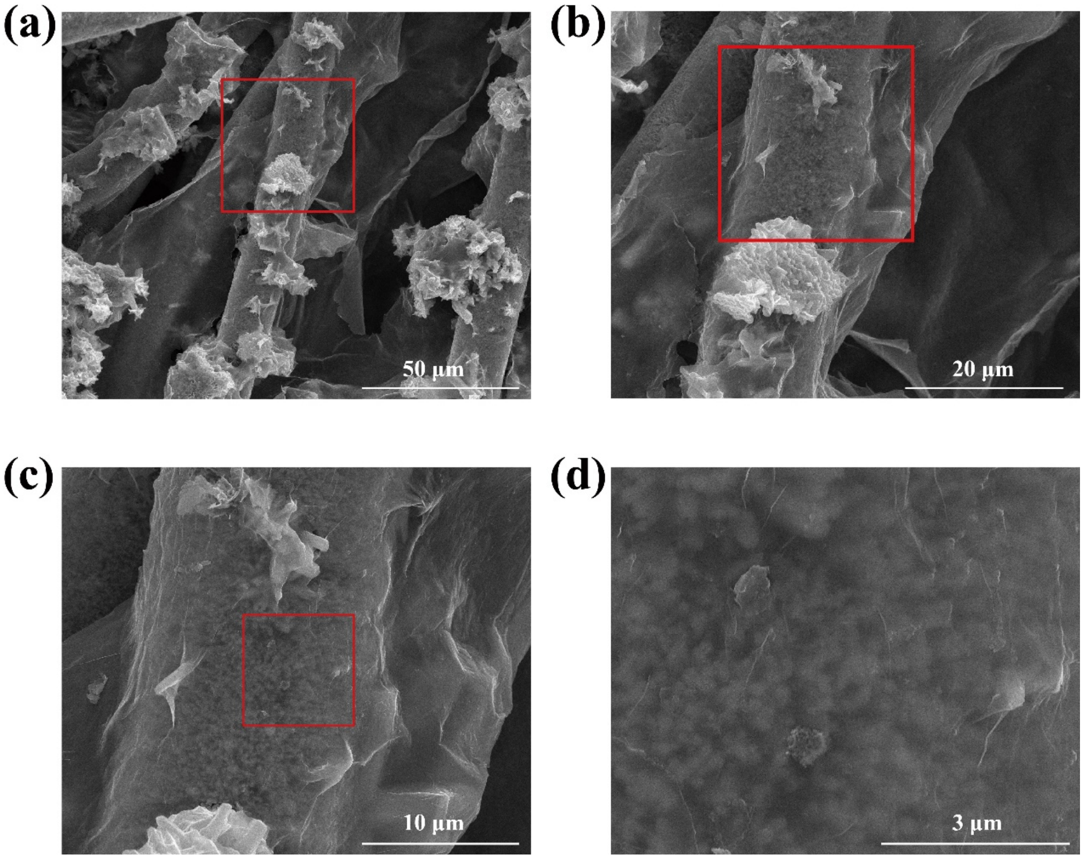

Figure S4. SEM images of PET/ZnO@MXene in different multiples (the red box is the enlarged part). (b) is the enlarged view of the red box in (a), (c) is the enlarged view of the red box in (b), and (d) is the enlarged view of the red box in (c).

Table S1. Comparison of different flexible sensing parameters between our sensor and some previously reported works.

Year	Material	Sensitivity (kPa^{-1})	Response /recovery time (ms)	Minimum pressure value	Ref.
2018	MXene/Reduced Graphene Oxide Aerogel	22.56	245 /212	245 Pa	[39]
2022	MXene/PVA composite gel	0.45	response (500)	2k Pa	[40]
2022	PET/MXene/Ag fiber	6.5	recovery (130)	/	[41]
2022	PET/MXene film	0.35	180 / 180	/	[42]
2022	PET/ZnO@MXene	53.22	150 / 100	60 Pa	This work

References

39. Ma, Y.; Yue, Y.; Zhang, H.; Cheng, F.; Zhao, W.; Rao, J.; Luo, S.; Wang, J.; Jiang, X.; Liu, Z.; Liu, N.; Gao, Y. 3D synergistical MXene/reduced graphene oxide aerogel for a piezoresistive sensor. *ACS Nano* **2018** *12*, *3209*.
40. Kallingal, N.; Maurya, M. R.; Sajna, M. S.; Yalcin, H. C.; Ouakad, H. M.; Bahadur, I.; Al-Maadeed S.; Sadasivuni, K. K. A highly sensitive wearable pressure sensor capsule based on PVA/Mxene composite gel. *3 Biotech* **2022** *12*, *171*.
41. Liu, H.; Yang, N.; Zhang, Q.; Wang, F.; Yan, X.; Zhang, X.; Cheng, T. A high-performance wearable Ag/$Ti_3C_2T_x$ MXene-based fiber sensor for temperature sensing, pressure sensing, and human motion detection. *IEEE Trans. Instrum. Meas.* **2022** *71*, *1*.
42. Ma, J.; Yang, K.; Jiang, Y.; Shen, L.; Ma, H.; Zhang, W.; Zhang J.; Zhu, N. Foot-scale MXene film of ultrathin electronic skin for wearable motion sensors. *Cell Rep. Phys. Sci.* **2022** *3*, 101013.
